# A proposed method of grading malaria chemoprevention efficacy

**DOI:** 10.1093/trstmh/trad042

**Published:** 2023-07-10

**Authors:** NJ White, C Bonnington, FH Nosten

**Affiliations:** Mahidol-Oxford Tropical Medicine Research Unit, Faculty of Tropical Medicine, Mahidol University, Bangkok 10400, Thailand; Centre for Tropical Medicine and Global Health, Nuffield Department of Medicine, University of Oxford, OX3 7LJ, UK; Malaria Consortium, London, UK; Shoklo Malaria Research Unit, Mahidol-Oxford Tropical Medicine Research Unit, Faculty of Tropical Medicine, Mahidol University, Bangkok 10400, Thailand

## Abstract

The efficacy and effectiveness of antimalarial drugs are threatened by rising levels of resistance and therefore require continuous monitoring. Chemoprevention is increasingly deployed as a malaria control measure, but there are no generally accepted methods of assessment. We propose a simple method of grading the parasitological response to chemoprevention (focusing on seasonal malaria chemoprevention) which is based on pharmacometric assessment (PARM).

## Introduction

Seasonal malaria chemoprevention (SMC) is the administration to children of treatment doses of antimalarial drugs at monthly intervals to suppress malaria in areas of highly seasonal malaria transmission. It is therefore a form of antimalarial chemoprophylaxis. For the most part SMC (formerly known as IPTc) has used a combination of amodiaquine and sulfadoxine-pyrimethamine (SPAQ) in children aged between 3 months and five years (3-59 months). SMC has been deployed across the Sahel region of Africa, a belt of intense seasonal malaria transmission. SPAQ is given monthly for 3-5 months [1]. The excellent results of large prospective evaluations, which overall showed an approximate 75% reduction in clinical malaria, resulted in adoption of SMC as policy by the World Health Organization in 2012 [2,3]. The adoption of this as policy was accompanied by a recommendation that methods be developed to assess and monitor the continued effectiveness of SMC. “Drug resistance monitoring and system evaluation should be supported or instituted, including systems to assess the number of breakthrough infections and their intervals from the last dose of SMC” [3]. But this recommendation was not followed, and today after hundreds of millions of doses of SPAQ have been deployed, there is still no accepted or recommended method of assessing this intervention. WHO also recommends other forms of chemoprevention in African children, notably intermittent prevention in infants (IPTi) which comprises giving treatment doses of SP delivered together with the EPI vaccines at 2,3 and 9 months of age. This has not been adopted widely. There is also no accepted or recommended method of assessing IPTi, or indeed any of the currently recommended forms of preventive antimalarial chemotherapy.

Meanwhile SMC deployment has been scaled up, and now it has spread across Africa. In 2022 WHO broadened substantially their original recommendations for SMC (which were confined to the Sahel) to other geographic regions in Africa (where drug resistance is worse) and removed restrictions on the number of monthly cycles or age [4,5]. Similarly, the recommendations for IPTi were loosened to include a broader age range and timing of drug administration. IPTi was renamed “perennial malaria chemoprevention” or PMC. The effectiveness of PMC depends on a drug that is failing in many malaria endemic regions. Resistance to sulfadoxine and pyrimethamine are widespread. But an earlier restriction for IPTi based on molecular markers of resistance indicative of high levels of resistance was removed [4] and no method to assess and monitor the continued effectiveness of this new PMC approach was suggested.

Funding to support malaria control is not unlimited. Clearly it is important for national control programmes, agencies and donors to know that the preventive interventions they are providing do work, and not to support interventions that are ineffective. Continued effective suppression of malaria by SMC justifies the considerable expense and efforts. But if the current interventions start to lose their effectiveness, this should be recognized quickly and alternative approaches should be deployed. Historically, the use of failing or useless antimalarial drugs has continued for many years and resulted in substantial associated human and financial costs [6]. This should not be allowed to happen again. All malaria interventions, and particularly those vulnerable to resistance, need robust methods of assessment so that their effectiveness can be monitored.

### Chemoprevention pharmacometrics

SMC differs fundamentally from the treatment of malaria. In SMC treatment doses of antimalarials are given to healthy children. Many of these children are infected with malaria parasites but at densities in blood which do not make them ill. In most cases they have already controlled their malaria infections. As a result, when they take an antimalarial drug, the therapeutic response is substantially better than in a symptomatic infection -which by definition has not been controlled by the host, and is usually associated with parasite numbers which are orders of magnitude higher [7,8]. Nevertheless, the drugs still have to provide some suppressive benefit during their long elimination phase in order to prevent new infections becoming patent (and potentially transmitting) until the next monthly treatment dose is given [9].

Pharmacometric Antimalarial Resistance Monitoring (PARM) describes a simple method of assessing chemoprevention antimalarial drug efficacy [8]. It relies primarily on dried filter paper samples from which measurements of both drug levels and qPCR parasite densities are made [8]. It does not require refrigeration or weekly follow-up. The key measure is the qPCR parasite density estimate at 28 days (i.e. before the next round of SMC). Filter paper qPCR on blood spots has a density limit of detection around 1-5 parasites/μL [10]. Filter paper sample drug measurement in a day 28 malaria positive sample allows the critical distinction of low drug exposure from drug resistance to be made. The same filter paper samples can be used for evaluation of molecular markers of drug resistance and distinction of reinfection from recrudescence. Taking blood slides at D28 to identify gametocytaemia is also informative if breakthrough rates are high (≥ M2, see below). An filter paper drug assay on day 7 provides additional information, and is particularly valuable for estimating exposure to more rapidly eliminated antimalarial drugs [8].

We propose a simple grading system to interpret the results of PARM and thus assess SMC effectiveness. This system should be modified and refined as monitoring results are obtained in different areas and with different drugs.

## Proposed simple grading system for malaria chemoprevention efficacy

The proposed grading depends primarily on the prevalence of filter paper based qPCR estimated parasitaemia 28 days after starting SMC. This does not discriminate between asexual and sexual stage parasites. This value is interpreted in relation to the simultaneously measured drug level(s). Detection of gametocytaemia by microscopy informs estimation of drug resistance selection pressures and transmissibility, but is not used in the proposed grading.

### M1

Even with fully sensitive malaria parasites and highly effective antimalarial medicines there are always some subjects who have low drug exposures (for various reasons including poor adherence, vomiting, pharmacokinetic variability, and low drug quality) [9]. An effective SMC or PMC should have less than 5% of recipients with a qPCR detectable malaria parasite densities at 28 days.

### M2

As chemoprevention begins to fail (either because of drug resistance or low drug exposures) the proportion of D28 detectable infections rises to between 5 and 10%. Gametocytaemia is present in a few subjects but densities are below the level of microscopy detection and unlikely to transmit. Suppression of further asexual stage multiplication by the next chemoprevention dose prevents generation of transmissible gametocyte densities.

### M3

The proportion of D28 detectable infections is between 10 and 20% and some subjects do have microscopy detectable gametocytaemia at D28 -which is presumably transmissible. This evidence of the selective pressure on drug resistance. Nearly all recurrent infections are new infections.

### M4

The proportion of D28 detectable infections is between 20 and 30% and many of these breakthrough infections now have microscopy detectable gametocytaemia. Some of the breakthrough infections are associated with illness. The majority of recurrent infections are still new infections.

### M5

The proportion of D28 detectable infections now exceeds 30% and many of these infections have microscopy detectable gametocytemia. Some recurrences are recrudescences but most recurrent infections are new infections and many are symptomatic.

### M6

Chemoprevention is completely ineffective. The proportion of D28 subjects who are parasitaemic is similar to that at baseline and there is little or no reduction in parasite prevalence or densities during the 28 days.

## Prevention of clinical illness

This proposed approach, based on basic pharmacometric principles [8], is designed to assess malaria chemoprevention effectiveness and inform policies. In antimalarial treatment therapeutic efficacy assessments malaria is detected actively and prevention of recurrent clinical illness is a secondary endpoint [11]. For chemoprevention, although effectiveness in preventing parasitaemia, reducing resistance selection, preventing transmission of drug exposed infections and preventing clinical malaria are all linked, it is prevention of illness that is the most important of these. This is the primary justification for deploying antimalarial chemoprevention. It is assumed that parasitological and clinical efficacy are closely linked, although in high transmission settings the majority of infections in older children are asymptomatic. Rising rates of parasitological failure are a harbinger of clinical failure. The grading system proposed would warrant concern at M2 and particularly at M3 levels, and consideration of policy change at M3 and above. The proportion of breakthrough infections which are symptomatic should be noted. Temperatures should be measured and a brief symptom enquiry made at the time of day 28 screening. If a significant proportion of breakthrough infections were symptomatic that would be additional cause for concern, prompting policy change. Whether the proportion of breakthrough infections which are symptomatic should be included in the grading system will require further study and evaluation.

## Discussion

SMC has been an excellent malaria control intervention across the Sahel and has provided a substantial benefit in reducing childhood malaria [12–16]. The three antimalarial drugs (amodiaquine and sulfadoxine-pyrimethamine in combination) are considered to retain good activity in this region, and the intense seasonality of malaria makes the SMC approach operationally feasible and thus cost-effective. In contrast, in East and Central Africa, where transmission is intense, malaria is often less seasonal and drug resistance is much worse, the benefit of unfettered deployment of SPAQ malaria chemoprevention is uncertain. In a responsible widescale deployment of an antimalarial (or indeed any anti-infective), intervention effectiveness must be measured. The method of assessment described here is robust but simple and operationally feasible, and it provides a quantifiable measure of effectiveness (encompassing efficacy and operational performance). It also informs on the cause of the chemoprevention failure. This grading is a proposal which will need refinement and improvement as information accrues. There are obviously risks associated with the newly expanded WHO chemoprevention policies and their implementation in areas with high levels of drug resistance, so information on chemoprevention effectiveness should be gathered urgently to guide responsible deployment.

SMC and PMC should only be deployed in areas where there is high transmission and a high burden of illness. In this context a substantial proportion of the target population is parasitaemic (by qPCR) at the beginning of the malaria transmission season. Obviously, if only a minority of subjects are parasitaemic before SMC is deployed and transmission is relatively low, or the study is done at the wrong time in relation to the transmission season, then there will be few breakthrough infections at D28. It could be concluded incorrectly that SMC was effective. Measuring the baseline prevalence addresses this question, although in some areas with intensely seasonal transmission the first round of SMC might be given just before the rapid rise in malaria prevalence.

In an attempt to justify continued use of the failing chloroquine it was once argued that control of parasitaemia was sufficient to justify antimalarial drug deployment, and it was suggested that clinical benefits could be dissociated from parasitological responses in malaria [17]. This is generally wrong, and that costly mistake should not be repeated. There are other effective drugs that can be used for SMC. If SPAQ chemoprevention is found to be effective it will reassure control programmes and donors that their efforts and investments are being rewarded. If it is found to be failing it must be substituted. Continued deployment of failing drugs will result in increased transmission and the associated morbidity and mortality and it will accelerate resistance. Continued measurement of chemoprevention effectiveness aims to prevent this from happening.

## Figures and Tables

**Figure:**
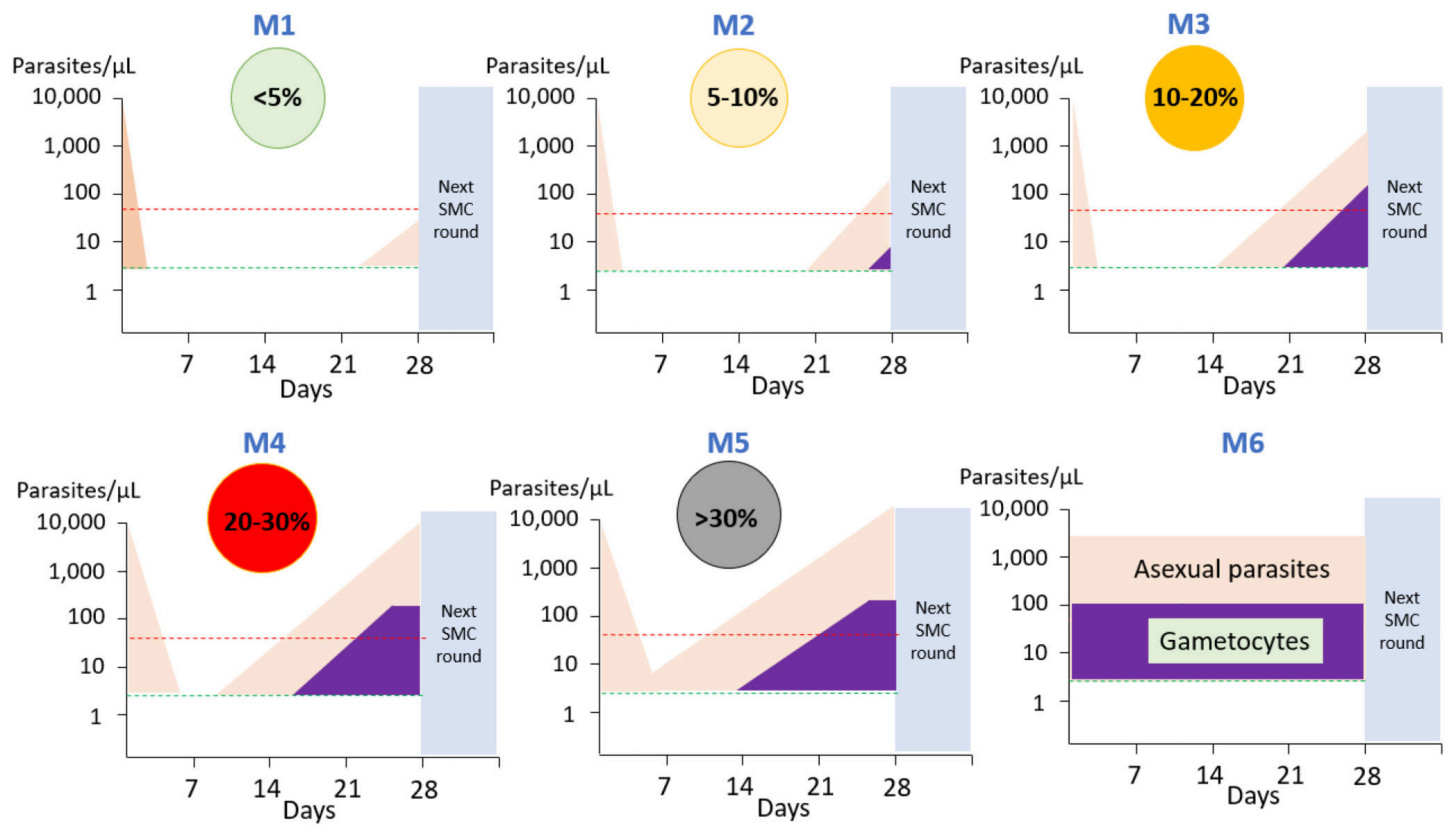
The proposed MORU grading of malaria chemoprevention effectiveness has six levels ranging from fully effective (M1) to completely ineffective (M6). Asexual malaria parasites (pink), if present, should be cleared by the SMC dose and reinfections suppressed for at least 28 days. The proportion of subjects who test qPCR positive for *P. falciparum* at 28 days provides the primary measure. At higher levels of SMC failure (≥M3) gametocytaemia (purple) is detectable by thick blood film microscopy and therefore likely transmissible. At very high levels of failure (≥M5) some infections detected at D28 are recrudescences. The red dashed line represents the lower level of microscopy detection and the green dashed line is the lower level of filter paper qPCR detection.
